# Modified turbidometric microassay for the measurement of sulfate in plasma and urine

**DOI:** 10.1016/j.mex.2024.102712

**Published:** 2024-04-18

**Authors:** Prasidhee Vijayakumar, Avis McWhinney, Yvonne A. Eiby, Paul A. Dawson

**Affiliations:** a Mater Research Institute, The University of Queensland, Woolloongabba, QLD, Australia; b Pathology Department, Mater Health Services, South Brisbane, QLD, Australia; c UQ Centre for Clinical Research and Perinatal Research Centre, The University of Queensland, Brisbane, Australia

**Keywords:** Sulfate assay, Barium chloride, Sulfatemia, Turbidometric microassay for measurement of inorganic sulfate

## Abstract

Sulfate is the fourth most abundant anion in circulation. Despite being an essential nutrient for healthy growth and development, sulfate is not routinely measured in clinical settings. In research settings, animal studies have shown that hyposulfatemia and hypersulfaturia are associated with adverse developmental outcomes. Those findings have increased interest in measuring plasma and urine sulfate levels. In this study, we describe a modified assay to measure sulfate in low volumes of plasma and urine.

• A streamlined microassay to measure sulfate levels using a microtiter plate format was developed.
• To determine the robustness of the assay, this method assessed reagent stability and concentrations, as well as absorbance at different wavelengths and following a range of incubation times.
• The optimized microassay was used to measure sulfate level in pig plasma and urine samples, which were compared to a validated ion chromatography method.

## Graphical abstract

**Figure:**
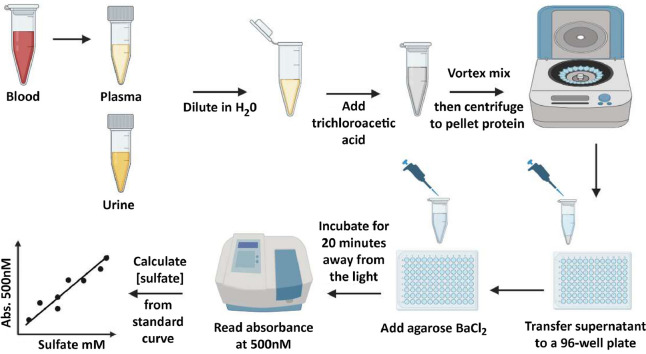

Specifications table

**Table:** 

Subject area:	Chemistry
More specific subject area:	Biochemical pathology
Name of your method:	Turbidometric microassay for measurement of inorganic sulfate
Name and reference of original method:	Simple, rapid, turbidometric determination of inorganic sulfate and/or protein. S G Jackson, E L McCandless. *Anal. Biochem.* 1978 Oct 15;90(2):802–8. doi: 10.1016/0003–2697(78)90,171–9
Resource availability:	Equipment: (1) Flat-bottom 96-well microtiter plates (Corning) (2) Microcentrifuge (3) Microplate spectrophotometer (Thermo Scientific™ Multiskan™ GO) Reagents: (1) Barium Chloride (Sigma-Aldrich Cat.# 202,738) (2) Agarose (Sigma-Aldrich Cat.# 05,066) (3) Trichloroacetic acid (Sigma-Aldrich Cat.# T6399) (4) Potassium sulfate (Sigma-Aldrich Cat.# 223,492) (5) Deionized H_2_O (Millipore)

## Background

Sulfate is an important nutrient in many cellular and physiological processes [[1], [2]]. Despite being the fourth most abundant anion in circulation, sulfate is not routinely measured in clinical settings. Animal models of hyposulfatemia display atypical phenotypes, including growth retardation, reduced fertility, seizures, behavioral abnormalities and osteochondrodysplasias [3], [4], [5]. Circulating sulfate level can be altered by genetic, environmental and physiological conditions [6], [7], [8], [9], [10]: (A) decreased by (i) loss-of-function mutations in the renal *SLC13A1* gene, (ii) certain drugs such as acetaminophen and (iii) low sulfate diet; and (B) increased (i) in maternal circulation during pregnancy; (ii) in patients with chronic kidney disease; and (iii) by high sulfate diet. These findings have led to increased interest in methodologies for measuring sulfate, particularly in plasma and urine.

Several methods for measuring sulfate in biological samples have been described, but many of those methods use toxic reagents [11] or require high-cost specialized equipment such as ion chromatography [12]. A relatively low-cost and low-hazard methodology is the turbidometric assay that uses BaCl_2_ agarose solution to precipitate sulfate [13]. However, the previously published turbidometric assay requires milliliter volumes of sample, which can exceed the volume of blood available from small laboratory animals in research settings or from infants in clinical settings.

In this study, we report a modified turbidometric microassay for measuring sulfate in low sample volumes. In addition, we tested a range of assay parameters, including reagent stability and reagent incubation times, as well as comparison between the turbidometric microassay and a validated ion chromatography method [12].

## Method details

### Plasma and urine samples

This study used samples from pigs up to 2 weeks of age. Whole blood was collected into a pediatric plasma separator tube, then gently inverted 5 times before centrifugal separation of the plasma at 9,000x*g* for 1.5 min in a microcentrifuge. The isolated plasma and a random urine sample were stored at −20 °C until assayed.

### Agarose barium chloride, trichloroacetic acid and potassium sulfate solutions

Agarose, BaCl_2_ and trichloroacetic acid were purchased from Sigma-Aldrich. Glassware was rinsed several times in deionized H_2_0 and then dried before preparing the reagents. A 0.5 % BaCl_2_ + 0.01 % agarose solution was prepared to a final volume of 50 ml using deionized H_2_O and a stock solution of 0.1 % agarose (dissolved by heating in deionized H_2_O) and then stored at room temperature from 1 to 11 weeks prior to use. An 8 % trichloroacetic acid solution was prepared in 50 ml deionized H_2_O. All reagents were stored away from light for up to 11 weeks. To prepare solutions with known sulfate levels, ranging from 0.05 to 2.0 mM, a stock 100 ml solution of 0.1 M K_2_SO_4_ (Sigma-Aldrich) was serially diluted in deionized H_2_0. These standard solutions were used to generate a standard curve.

### Sample preparation

Plasma and urine samples were diluted in deionized H_2_O at ratios of 1:1 and 1:49, respectively. In 1.5 ml tubes, 120 µl of 8 % trichloroacetic acid were added to 110 µl K_2_SO_4_ standards and the diluted plasma and urine samples. Samples were vortex mixed for 5 s and then centrifuged at 16,000x*g* for 3 min at room temperature. Supernatants were transferred to fresh 1.5 ml tubes and centrifuged at 16,000x*g* for 3 min at room temperature. Supernatants from this second round of centrifugation were used in the sulfate microassay.

### Sulfate microassay

Duplicate 80 µl aliquots of supernatants containing plasma, urine, K_2_SO_4_ standards or deionized H_2_O (control no sulfate) were transferred to a 96-well flat bottom microtiter plate. To each well, 20.87µl agarose BaCl_2_ was added and mixed by stirring with the pipette tip, rather than pipetting up and down, to avoid bubbles. The microtiter plate, covered in foil to shield from light, was incubated at room temperature for 20 min. The absorbance of each well was then measured at 500 nM using a microplate spectrophotometer (Thermo Scientific™ Multiskan™ GO). Sulfate concentration in the plasma and urine samples was then calculated from the linear plot of absorbance values for each of the K_2_SO_4_ standards.

## Method validation

### Modified low volume sulfate assay

In this study, we modified a previously published turbidometric assay [13] for measuring sulfate by reducing the sample volume from 1.1 ml to 80 µl using a microtiter plate and a microplate spectrophotometer. Considering that plasma makes up approximately 55 % of blood [14], and accounting for the dilution of plasma (1:1) and urine (1:49) used in this assay, the minimum volumes of whole blood and urine required for this microassay are 100 µl and 2.2 µl, respectively.

### Linearity of sulfate detection

**Fig. 1:**
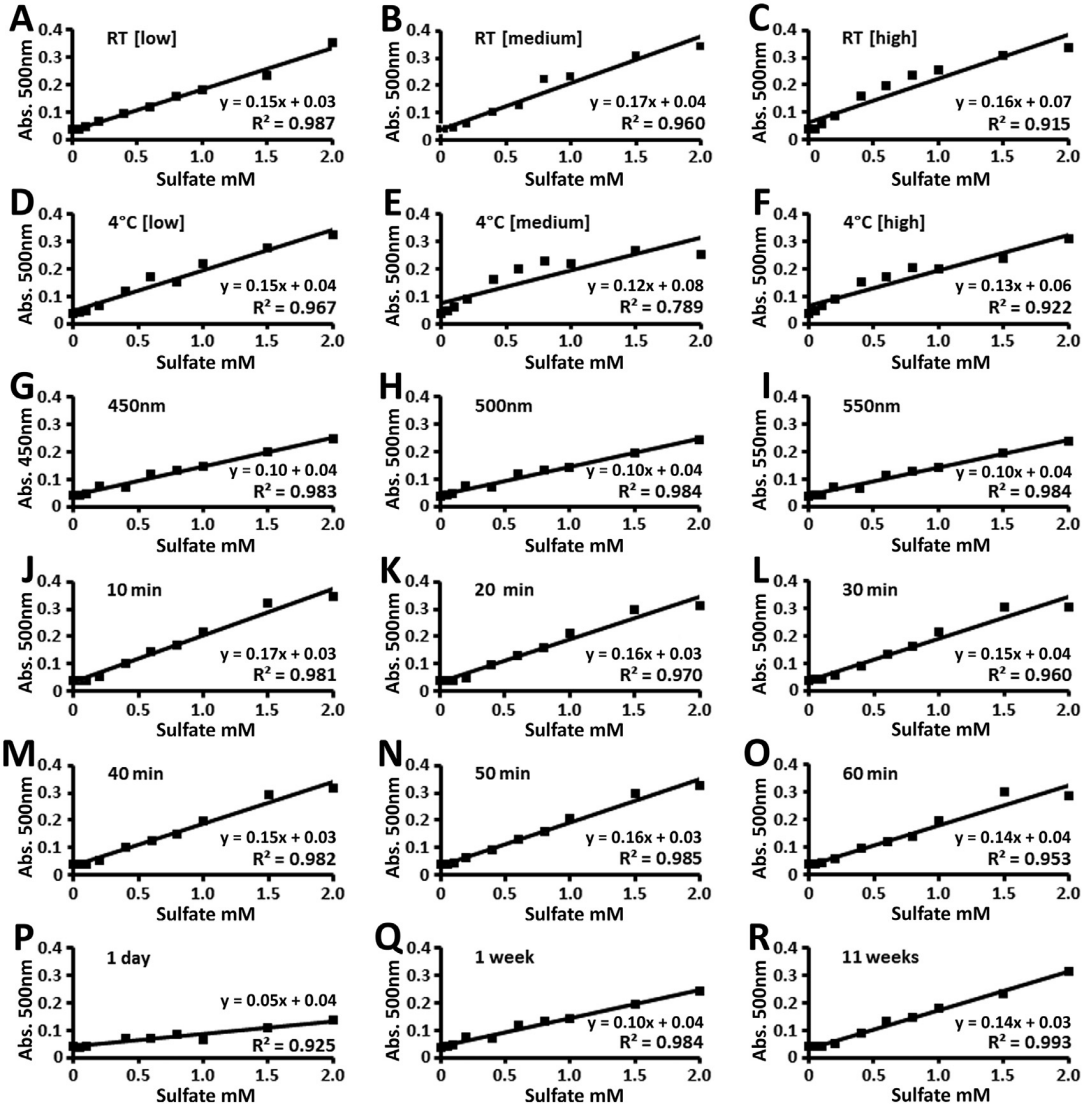
Linearity of sulfate detection using K_2_SO_4_ standards to test reagent concentrations and stability, as well as absorbance at different wavelengths and incubation times in the modified turbidometric microassay. Data are representative of at least 10 independent experiments and the within run imprecision coefficient of variation (CVa) was <1.0 %. A–F) Assays used BaCl_2_ agarose solution at low (0.25 % BaCl_2_ + 0.005 % agarose), medium (0.5 % BaCl_2_ + 0.01 % agarose) or high (1.0 % BaCl_2_ + 0.02 % agarose) concentrations that were stored at room temperature (RT) or 4 °C for 1 week. G–I) Using the medium concentration of BaCl_2_ agarose solution stored at RT for 24hr, the absorbance values were measured at 450 nm, 500 nm or 550 nm. J–O) Using the conditions from panel H, absorbance values were measured at timed intervals, from 10 to 60 min, after mixing the BaCl_2_ agarose solution with K_2_SO_4_ standard solutions. P–R) Samples were assayed with the parameters used in panel K, following storage of the BaCl_2_ agarose solution at RT for up to 11 weeks.

To determine whether the previously published assay [13] could be further improved, we tested a range of assay parameters (Fig. 1). We show that both low and high BaCl_2_ agarose concentrations stored at either room temperature or 4 °C gave similar linear responses to the previously published (medium) concentration stored at room temperature (Fig. 1A–F). In addition, the linearity of sulfate detection was similar when 450 nm, 500 nm (used in previously published method) or 550 nm wavelengths were applied (Fig. 1G–I). We next tested a range of incubation times post-mixing the sample and BaCl_2_ agarose reagent (Fig. 1J–O). Linearity of detection was similar between all incubation times, indicating the microassay can be completed at a minimum time of 10 min, which is less than the previous method that used an incubation time of 35–40 min. Stability of BaCl_2_ agarose was also tested, showing that this reagent is stable at room temperature up to at least 11 weeks post-preparation, with higher linear response and slope at 1 and 11 weeks when compared to 1 day (Fig. 1P–R).

### Recovery of sulfate

Using assay parameters shown in Fig. 1, the limit of detection (LOD=3.3σ/S, 0.097 mmol/L) and quantification (LOQ=10σ/S, 0.294 mmol/L) were calculated from 3.3 to 10 times, respectively, the SDs of responses, where the slope of the calibration curve (S)=0.17 and the SD of y intercepts of regression lines (σ)=0.005. These LOD and LOQ values are low enough for the detection and quantification of sulfate in diluted plasma and urine samples from laboratory animals, including pigs and mice which have plasma and urine sulfate levels in the millimolar range [3,15]. To calculate the recovery of sulfate in this turbidometric microassay, we added known amounts of sulfate to pig plasma and urine followed by dilution and processing as described above. The average sulfate recovery ranged from 45 to 66 % for plasma and 58–66 % for urine spiked with 0.8–1.5 mmol/L K_2_SO_4_ and 25–100 mmol/L K_2_SO_4_, respectively (Table 1). This finding suggests that the turbidometric microassay underestimates the sulfate level in plasma and urine by at least 34 %. We found that to be the case when we compared sulfate levels measured with the turbidometric microassay and an ion chromatography method [12]. The turbidometric microassay consistently yielded sulfate levels that were 32 % and 33 % lower in pig plasma (*n* = 33) and urine (*n* = 5) samples, respectively, when compared to the ion chromatography method. Taken together, these findings indicate that a 1.3-fold factor should be used when calculating plasma and urine sulfate levels with the turbidometric microassay.

**Table 1:** Recovery of sulfate using the turbidometric microassay.

*Sample	K_2_SO_4_ spike (mmol/L)	Recovery sulfate% (Mean±SEM)
Plasma	0.8	66±18
	1.0	52±11
	1.5	45±9
Urine	25	66±15
	50	58±14
	100	63±22

*Plasma and urine samples were spiked with known concentrations of K_2_SO_4_ and then diluted with H_2_0 1:1 and 1:50, respectively, before being processed and assayed.

In this study, we report a modified assay for measuring sulfate level in low sample volumes. This is particularly relevant to research studies that require sulfate testing in multiple blood and urine samples from laboratory animals. In addition, we reduced the assay incubation time and showed that the assay reagents can be stored for at least 11 weeks at room temperature, avoiding the need for freshly prepared solutions and cold storage. We also show that the microassay underestimates sulfate concentration by approximately 33 %. Accordingly, we recommend including a 1.3x factor when calculating plasma and urinary sulfate level.

## Ethics statements

All procedures were approved (2022/AE000846) by the University of Queensland Animal Ethics Committee. This study used plasma and urine samples from both male and female pigs to compare methodology. Data were not compared among pigs.

## CRediT authorship contribution statement

**Prasidhee Vijayakumar:** Conceptualization, Methodology, Validation, Formal analysis, Investigation, Writing – original draft, Visualization. **Avis McWhinney:** Investigation, Resources, Writing – review & editing. **Yvonne A. Eiby:** Conceptualization, Methodology, Investigation, Resources, Writing – review & editing, Supervision, Project administration, Funding acquisition. **Paul A. Dawson:** Conceptualization, Methodology, Validation, Investigation, Resources, Writing – review & editing, Visualization, Supervision, Project administration, Funding acquisition.

## Declaration of competing interest

The authors declare that they have no known competing financial interests or personal relationships that could have appeared to influence the work reported in this paper.

## Data Availability

Data will be made available on request.
